# Molecular marker assisted breeding and genome composition analysis of Zhengmai 7698, an elite winter wheat cultivar

**DOI:** 10.1038/s41598-017-18726-8

**Published:** 2018-01-10

**Authors:** Chun-xin Li, Wei-gang Xu, Rui Guo, Jian-zhou Zhang, Xue-li Qi, Lin Hu, Ming-zhong Zhao

**Affiliations:** Molecular Breeding Laboratory, Wheat Research Institute, Henan Academy of Agricultural Sciences, Zhengzhou, 450002 Henan China

**Keywords:** Plant breeding, Plant breeding

## Abstract

Zhengmai 7698 is an elite winter wheat variety widely cultivated in the Southern regions of the Yellow-Huai River Valley of China. Here, we report the molecular markers used for breeding Zhengmai 7698 and the genome composition of this cultivar revealed using genome-wide SNPs. A total of 26 DNA markers derived from the genes controlling gluten protein quality, grain hardness, flour color, disease resistance, or pre-harvesting sprouting resistance were used during breeding. Consequently, Zhengmai 7698 had strong gluten, high grain hardness index, white flour color, and high levels of resistance to powdery mildew, stripe rust infections, and pre-harvesting sprouting. Using genome complexity reduction, 28,996 high-quality SNPs distributed on 21 wheat chromosomes were identified among Zhengmai 7698 and its three parental lines (4B269, Zhengmai 9405 and Zhoumai 16). Zhengmai 7698 shared 12,776, 14,411 and 16,085 SNPs with 4B269, Zhengmai 9405 and Zhoumai 16, respectively. Thus, the contributions of 4B269, Zhengmai 9405 and Zhoumai 16 to the genome of Zhengmai 7698 were comparable. Interestingly, Zhengmai 7698 had 307 unique SNPs that are absent in all three parents. We suggest that molecular markers facilitate selection of a wheat cultivar with multiple elite traits. Analysis of genome composition with SNPs may provide useful clues for further dissecting the genetic basis of improved wheat performance.

## Introduction

Wheat, one of the three major cereal crops in the world, is a staple food for approximately 40% of the world’s population. Wheat consumption has been increasing in recent years, which is a great challenge to wheat breeders^1^. Therefore, breeding high-yielding, and high quality wheat cultivars that have resistance to multi-diseases and cost-effective is crucial for future wheat production. Several problems have been identified in the current wheat breeding including lack of understanding contribution of each parental line to their progenies, the genes underlying important agronomic traits as well as lack of cultivar-specific markers for identification.

However, new biology techniques can be applied to address these problems and gain a better understanding of the genetic contribution of parental lines to their offspring. Furthermore, bio-technologies can be applied to rapidly, accurately and consistently identify the authenticity of cultivars, improve breeding procedures, and protect the legal rights of breeders and enterprises.

For a long time, geneticists have attempted to elucidate the genetic basis of founder parents and some popular wheat cultivars. At present, genetic analyses of wheat cultivars in China are primarily based on simple sequence repeat (SSR) markers. For example, Li *et al*.^2^ used 657 pairs of SSRs to analyse the genetic composition of sister and parental lines of Bainong AK58 thereby determining the contribution of each parent to Bainong AK58. Zou *et al*.^3^ and Li *et al*.^4^ used SSR and DArT markers to analyse the origin of genetic components of Zhoumai 23 and Chuanmai 104 and discovered that some alleles are related to superior traits. Li *et al*.^5^ used SSR markers to study the distribution of chromosomal segments of the founder parent Orofen in its subsequent wheat progenies and to study its associated agronomic traits, including spikelet number per panicle and yield. Xiao *et al*.^6^ analysed the genetic structure of Zhou 8425B-derived wheat cultivars with DArT and SSR markers and identified stripe rust resistance genes. Xu *et al*.^7^ studied the transmission of 1BL/1RS translocation lines from Lovrin 10-derived cultivars to their offspring. These studies provide a theoretical basis for the promotion of target cultivars and utilization of founder parents for breeding. However, the aforementioned studies primarily used SSRs as markers, which are relatively low (<1000) in number and may not be able to provide desirable genomic coverage. Single nucleotide polymorphism (SNP) markers are unlimited in number therefore, can effectively bridge this gap. However, studies using high-density SNPs to examine the genomic origin of widely grown cultivars and the genetic contribution of their parental lines are lacking. Zhengmai 7698 is a wheat cultivar with the pedigree Zhengmai 9405/4B269//Zhoumai 16. Currently, it is one of the main commercial varieties in the Huanghuai wheat production region of China. Zhengmai 7698 yield could reach 11,287.5 kg/ha in a test field spot^8^ thus setting a new yield record for high-quality strong-gluten wheat cultivars in China. In recent years, this cultivar has also become an important wheat breeding parent in the Huanghuai wheat production area. However, the differences in genomic composition between Zhengmai 7698 and its parents, Zhengmai 9405 (ZM025882, National Crop Gene Bank, Beijing, China), 4B269, and Zhoumai 16 (ZM025894, National Crop Gene Bank, Beijing, China), still remain unclear. In addition, systematic studies of specific detection markers associated with superior agronomic traits of Zhengmai 7698 are lacking.

In this study, the specific-locus amplified fragment sequencing (SLAF-seq) method was used to identify high-density SNPs to analyse the genetic composition of Zhengmai 7698 and its parents at various genomic loci and determine the genetic contribution of each parent. In addition, SNPs specific to Zhengmai 7698 were identified, and some important genes controlling agronomic traits in this cultivar were analysed using closely linked or functional markers for those agronomic traits. The results will provide a reference for high yield wheat breeding and support for the utilization of Zhengmai 7698.

## Results

### Molecular markers for Zhengmai 7698

During the breeding of Zhengmai 7698, molecular markers were used to accelerate selection of certain phenotypic traits that are typically more difficult to select and for traits that can be pyramided thereby improving selection accuracy. The combined application phenotypic and molecular marker selection ensured successful pyramiding of three powdery mildew (Pm) resistance genes (*Pm2*, *Pm4b*, and *Pm8*), two stripe rust (Yr) resistance genes (*YrZH84* and *Yr9*), two pre-harvest sprouting (PHS) resistance genes (*PHS1* and *PHS4A*), and certain high-quality genes (Table 1), which effectively improved disease resistance, and the quality of Zhengmai 7698. In the F_3_–F_6_ generations of the selection processes, seven superior genetic markers in the parents were used (genes underlined by straight line in Table 1) to monitor the progress of gene pyramiding in hybrid offspring (e.g., single plant and line), and in the F7 and subsequent stable generations, and 19 newly developed genetic markers were progressively added (genes underlined by wavy line in Table 1).

**Table 1:** Names and their primer sequences of markers used in the marker assisted selection of Zhengmai 7698 (the label √ means the existence of that gene).

**Traits**		**Gene**	**Zhengmai**	**Parents**			**Markers and their primers**			**Reference**
**Superior traits**	**High quality gene**		**7698**	**4B269**	**Zhengmai 9405**	**Zhoumai 16**	**Marker**	**Forward primers (5′-3′)**	**Reverse primers (5′-3′)**	
High molecular weight glutenin subunits		***Ax1***	√	√			UMN19	CGAGACAATATGAGCAGCAAG	CTGCCATGGAGAAGTTGGA	^10^
		*Ax-null*			√	√	UMN19	CGAGACAATATGAGCAGCAAG	CTGCCATGGAGAAGTTGGA	^10^
	***Bx7***	***Bx7***	√	√	√	√	Bx7	CACTGAGATGGCTAAGCGCC	GCCTTGGACGGCACCACAGG	^11^
	***By8***	***By8***		√	√		ZSBy8	TTAGCGCTAAGTGCCGTCT	TTGTCCTATTTGCTGCCCTT	^12^
		*By9*	√			√	ZSBy9a	TTCTCTGCATCAGTCAGGA	AGAGAAGCTGTGTAATGCC	^12^
		*Dx2*				√	UMN25	GGGACAATACGAGCAGCAAA	CTTGTTCCGGTTGTTGCCA	^10^
		***Dx5***	√	√	√		Dx5	CGTCCCTATAAAAGCCTAGC	AGTATGAAACCTGCTGCGGAC	^13^
		***Dy10***	√	√	√		UMN26	CGCAAGACAATATGAGCAAACT	TTGCCTTTGTCCTGTGTGC	^10^
		*Dy12*				√	UMN26	CGCAAGACAATATGAGCAAACT	TTGCCTTTGTCCTGTGTGC	^10^
Grain hardness		*Pinb-D1a*	√	√	√		Pinb-D1a	ATGAAGACCTTATTCCTCCTA	CTCATGCTCACAGCCGCC	^14^
		*Pinb-D1b*				√	Pinb-D1b	ATGAAGACCTTATTCCTCCTA	CTCATGCTCACAGCCGCT	^14^
Lipoxygenase		*TaLox-B1a*	√		√		LOX16	CCATGACCTGATCCTTCCCTT	GCGCGGATAGGGGTGGT	^15^
		*TaLox-B1b*		√		√	LOX18	ACGATGTGAGTTGTGACTTGTGA	GCGCGGATAGGGGTGC	^15^
Yellow pigment content		*Psy-A1a*				√	YP7A	GGACCTTGCTGATGACCGAG	TGACGGTCTGAAGTGAGAATGA	^16^
	***Psy-A1b***	***Psy-A1b***	√	√	√		YP7A	GGACCTTGCTGATGACCGAG	TGACGGTCTGAAGTGAGAATGA	^16^
		*Psy-B1a*	√		√	√	YP7B-1	GCCACAACTTGAATGTGAAAC	ACTTCTTCCATTTGAACCCC	^17^
	***Psy-B1b***	***Psy-B1b***		√			YP7B-1	GCCACAACTTGAATGTGAAAC	ACTTCTTCCATTTGAACCCC	^17^
		*Psy1-D1g*		√	√		YP7D-1	TCCGACACCATCACCAAGTTCC	CGTTGTAGGTTTGTGGGAGT	^18^
Polyphenol oxidase activity		*PPO-A1a*		√		√	PPO18	AACTGCTGGCTCTTCTTCCCA	AAGAAGTTGCCCATGTCCGC	^19^
	***PPO-A1b***	***PPO-A1b***	√		√		PPO18	AACTGCTGGCTCTTCTTCCCA	AAGAAGTTGCCCATGTCCGC	^19^
	***PPO-D1a***	***PPO-D1a***	√			√	PPO16	TGCTGACCGACCTTGACTCC	CTCGTCACCGTCACCCGTAT	^20^
		*PPO-D1b*		√	√		PPO29	TGAAGCTGCCGGTCATCTAC	AAGTTGCCCATGTCCTCGCC	^20^
Powdery mildew resistant		*Pm2*	√	√	√	√	Pm2	AGCTGTTTGGGTACAAGGTG	GCCATCGTTTTCTACTAG	^21^
		*Pm4b*	√		√	√	Pm4b	GTGGTGTATCAAATGTCATCAGTACTAC	TCCAGTGACCCCATCTGCTCATAC	^21^
		*Pm8*	√	√	√	√	Pm8	GGAGACATCATGAAACATTTG	CTGTTGTTGGGCAGAAAG	^21^
Yellow rust resistant		*Yr9*	√	√		√	Xgwm582	AAGCACTACGAAAATATGAC	TCTTAAGGGGTGTTATCATA	^22^
		*YrZH84*	√		√		Xcfa2040	TCAAATGATTTCAGGTAACCACT	TTCCTGATCCCACCAAACAT	^23^
Pre-harvest sprouting		*PHS1*	√	√		√	PHS1	GGTGGAACAGATGCAACTAAAGG/ GGTGGAACAGATGCAACTAAAGA	GTGAGTGTTATATGAAACTAATGATCCATT	^24^
resistant		*PHS-4AL*	√	√			PHS-4AL	TGGAGTCTGAAAGCATTCGA/TGGAGTCTGAAAGCATTCGG	TCCATGCATCATAGGAAAACA	^25^

To select for three specific traits, molecular markers were combined with phenotyping. Molecular markers of stripe rust resistance were combined with field inoculation of strong virulent races (e.g., CY32, CY33, G22-9, G22-14, SU4, SU5) for resistance identification, and stripe rust resistance was obtained by pyramiding *Yr9* and *YrZH84*. The powdery mildew resistance genes were combined with natural infection in the Powdery Mildew Disease Nursery, the Yuanyang test site of the Wheat Research Institute, Henan Academy of Agricultural Sciences, where high incidence of powdery mildew was observed. Powdery mildew resistance was obtained by pyramiding the genes *Pm2*, *Pm4b*, and *Pm8*. The analysis of markers for pre-harvest sprouting resistance genes revealed that Zhengmai 7698 carried the major pre-harvest sprouting resistance genes *PHS1* and *PHS*-4AL. In addition, a set panel of molecular markers for quality-related traits was used in the final selection of advanced elite breeding lines. Detailed results of gene pyramiding for quality traits in Zhengmai 7698 are shown in Table 1. To confirm genes pyramiding results, we compared the traits, including yields, quantity, powdery mildew resistance, strip rust resistance and pre-harvest sprouting resistance, of Zhengmai 7698 and its three parents, which showed that Zhengmai 7698 had successfully pyramided the most superior traits from its parents (Table 2).

**Table 2:** Phenotypes of part agronomic traits in Zhengmai 7698 and three parents.

Cultivar	PM	YR	PHS	WGC	ST(min)	Yield(kg/ha)
Zhoumai 16	2	2	41.32%	25.90%	2.1	8513.99
Zhengmai 9405	3	2	44.21%	37.40%	10.1	7679.70
Zhengmai 7698	2	2	18.29%	32.80%	7.9	8820.67
4B269	2	4	18.16%	31.10%	9.2	7810.70

Abbreviations: PM, Powdery mildew; YR, Yellow rust; PHS, Pre-harvest sprouting; WGC, Wet gluten contents; ST, stability time. To PM and YR, the number 0–1 represent high resistance; 2 represents medium resistant; 3 means medium susceptible; 4 means high susceptible.

Based on the marker assisted selection (MAS) practice of Zhengmai 7698, we proposed a method named marker monitoring program, which a marker panel was used to monitor the target genes pyramiding in segregating populations that were selected for their excellent traits performance. To concrete protocol, the markers were used to detect the genotyping of the plants that already been selected for their phenotype. The plants with target genes were saved as next generation plans. There plants that have superior traits performance (according main traits of breeding objective) than the plants having target genes were also saved as next generation plans. This markers detection will not stop until all the target loci are homozygous. This selection protocol usually can be used in F2, F3, F4 and F5 population. However, the F2 is the key generation, which decides the genetic background and population size for following generations.

We try this protocol in two crossing, Zhengmai 7698/Gaoyou 2018 and Jichuang 2/Xinmai 26//08H107, with wheat quantity markers (Table 1). Firstly, we selected 300 plants from two crossing respectively. The marker panel (Table 1) was used to detect the distribution of the quantity genes. The results showed that 12 (Zhengmai 7698/Gaoyou 2018) and 23 (Jichuang 2/Xinmai 26//08H107) plants, pyramided all target genes from their parent. After comparing the with other plants (without target genes) in phenotypes of traits, such as tiller, yield, and maturity time etc., we finally chosen 22 and 35 plants as F3 from two crossing. The size of plants, pyramiding target genes, is determined by the linkage distance of the maker and distribution of the gene in parents.

### Genetic structure analysis of Zhengmai 7698 and its parental lines

**Figure 1:**
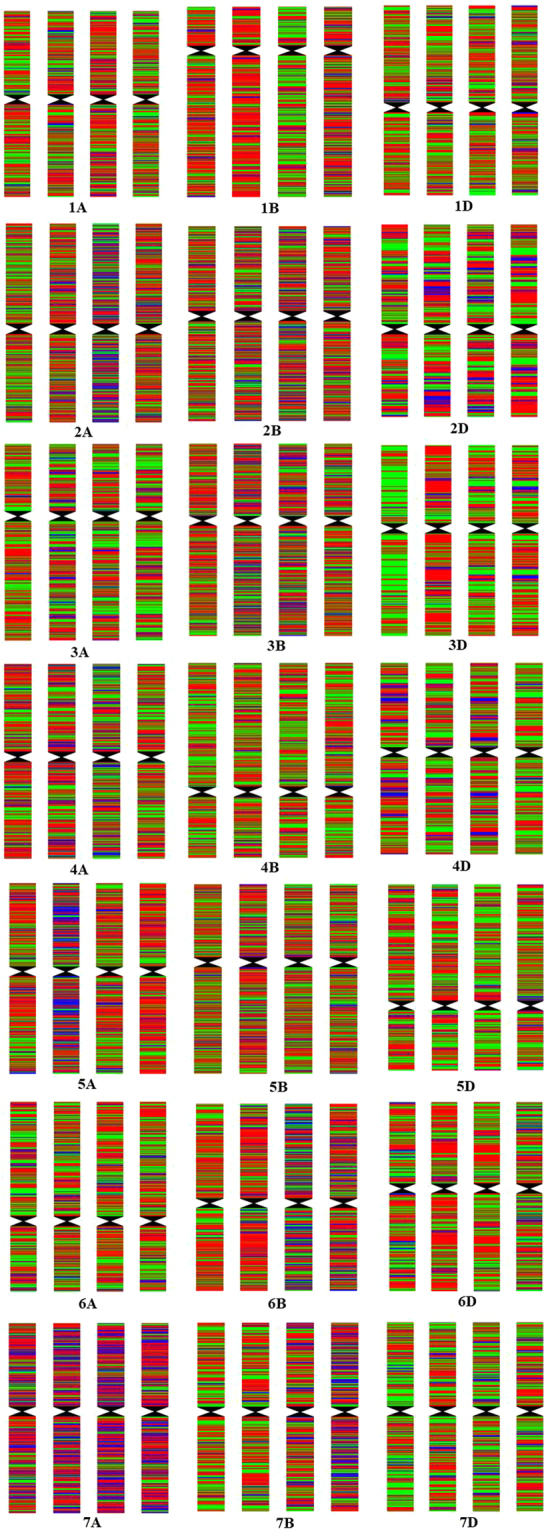
Genotypic patterns of the samples in the Zhengmai 7698 and its three parents cross based on SNP loci on 21 chromosomes. Sample name, start from left to right, in each genotypic pattern are Zhengmai 7698, 4B269, Zhengmai 9405 and Zhoumai 16.

Based on the predicted restriction enzyme digestion rates, *Rsa*I had a digestion efficiency of 94.83%, thus was selected for digestion. An average Q30 score for these sequences was 95.26%, and the average GC content was 45.71%. All the sequences results were aligned and the SNPs were called using Chinese Spring reference sequence genome. A total of 71,516 SNPs was identified. Among them 28,996 are high-quality SNPs after filtering the SNP by setting a minor allele frequency (MAF) >0.05 and integrity >0.8 (Table 3). Among these high quality SNP loci, 4,677 were the same in Zhengmai 7698 and the three parental plants. By comparing the sequences between Zhengmai 7698 and parental lines, a genotype map of Zhengmai 7698 was constructed based on the physical locations of different SNP loci on the chromosomes (Fig. 1).

**Table 3:** Distribution of SNP loci and specific loci among Zhengmai 7698’s chromosomes.

Chromosome	SNP loci	Specific SNP loci	Chromosome	SNP loci	Specific SNP loci	Chromosome	SNP loci	Specific SNP loci
1A	1209	28	1B	1190	6	1D	1382	8
2A	2442	17	2B	2241	7	2D	578	9
3A	1095	9	3B	2255	15	3D	299	5
4A	1575	14	4B	1296	18	4D	874	8
5A	1414	8	5B	2778	28	5D	1342	18
6A	1121	5	6B	1467	1	6D	992	19
7A	1291	72	7B	1026	7	7D	1129	5
Total	10147	153		12253	82		6596	72

Of the 28,996 high-quality SNPs, 12,776 Zhengmai 7698 SNPs were identical to those in 4B269, 14,411 were identical to those in Zhengmai 9405, and 16,085 were identical to those in Zhoumai 16. The genetic contributions of Zhengmai 9405, 4B269, and Zhoumai 16 were 49.70%, 44.06%, and 55.50%, respectively. Assuming that the sum of all genetic contributions from the parents was 100%, the relative genetic contributions of the three parents were 33.3%, 29.52%, and 37.18%, respectively (Table S1); thus, the three parents made the similar contribution to Zhengmai 7698.

However, the SNP contribution was significantly different among chromosomes with amplified genetic differences from different parents. In particular, in the chromosome Group A, 40.88% and 48% of genetic loci originated from the 5A and 7A of Zhengmai 9405, and 41% loci were from the 4A of Zhoumai 16. As for the remaining chromosomes, their SNP contribution diversity from each of the three parents did not exceed 10%. The genetic transmission rates of Zhengmai 9405, 4B269, and Zhoumai 16 to the Group A chromosomes in Zhengmai 7698 were 35.70%, 29.03%, and 35.27%, respectively, which were similar to the whole genetic transmission rates. Among the Group B chromosomes, only 6.09% of the 5B chromosome components were from 4B269, while the genetic transmission rate of other two parents was 46.96% each. Transmissibility of the three parents to the Group B chromosomes of Zhengmai 7698 was 32.46% (Zhengmai 9405), 26.87% (4B269), and 40.67% (Zhoumai 16). And the contributed of the three parents to the Group D chromosomes was 33.03% (Zhengmai 9405), 31.43% (4B269), and 35.54% (Zhoumai 16) (Table S1).

### Gene annotation and functional analysis of genomic SNP loci in Zhengmai 7698

Annotation of the 28,996 SNPs using the public GO database associated 521 SNPs to 175 specific genes, of which 146 were functionally annotated (Fig. S1). These predicted genes predominantly can be associated with following functions: protein kinases, membrane proteins, cell division, nucleic acid binding site, substance transport, phosphorylation, and electron transfer. Their products were either intermediates or substrates of various physiological and biochemical reactions that are involved in various stages of plant physiological development. However, when enrichment those genes in Kyoto Encyclopedia of Genes and Genomes (KEGG), we found those genes involved in 29 pathways (Fig. S2), in which 8 significant enrichment pathways(Table S2) were considered reliable.

### Predictive analysis on the origin and function of Zhengmai 7698-specific SNP loci

Comparison of the Zhengmai 7698 genotype with those of its parents showed that a total of 307 SNP loci (Table 3) were specific to Zhengmai 7698 accounting for 1.1% of all SNPs. After these loci annotation (www.geneontology. org), we found that there main functions were serine/threonine protein kinases, oxidoreductase, DNA binding sites, and transmembrane transport proteins (Table S3). To discovering the gene enrichment in pathway, 307 SNP loci were analysed with KEGG. The results showed that specific SNP loci were enriched in 20 pathways, in which only one, RNA transport, was significance (Fig. S3).

## Discussion

Zhengmai 9405, one of the parents of Zhengmai 7698, is characterised as a high grain-quality and strong-gluten cultivar. 4B269 is a high-quality, multi-resistant founder parent developed by our breeding group. The last parent, Zhoumai 16, is a high-yielding cultivar with moderate to low quality gluten. Molecular identification of Zhengmai 7698 indicated partial gene pyramiding for pre-harvest sprouting resistance, disease resistance, and high-quality alleles in the three parents. Based on genotyping of 28,996 SNPs, the actual genetic contribution of Zhengmai 9405, 4B269, and Zhoumai 16 were found to be 49.70%, 44.06%, and 55.50%, respectively.

To this study, a large segment originating from any of the three parents was not found (Fig. 1), indicating full recombination of the three parental genomes in the successive progeny selection processes. Therefore, the three parents were fully involved in the formation of the Zhengmai 7698 genome. The differences in genetic contribution rates among the parents were the result of directional selection by breeders. However, Zhengmai 7698 outperformed its parents in yield trait to a certain extent. This phenomenon might be due to gene interactions between yield-related loci from different parents or genetic variation caused by the 307 mutant SNPs.

Previously, some studies have reported that entire chromosomes are derived from a single parent. In many of these cases, the low density of markers affected the accuracy of the results because only single markers represented entirechromosomes^2–5^. In this study, 28,996 high-quality SNPs were identified using SLAF-seq technology, which resulted in reasonable marker coverage of each chromosome. We did not see a single chromosome that was contributed by single parents, suggested that marker coverage is appropriate in this study and all parents contributed to each chromosomes of Zhengmai 7698 genome. The phenotype of (Table 2) Zhengmai 7698 showed that it combined the profitable traits of the three parents. The molecular marker detective results also indicated that partial gene of the three parents, pre-harvest sprouting resistance, disease resistance and high-quality alleles, were pyramided in the Zhengmai 7698.

Overall, the differences of the parents in genetic contribution were not significant, in which Zhoumai 16 had a slightly larger genetic contribution as a second hybridization parent. In terms of specific chromosomes, the genetic contributions of the three parents were also similar. Only chromosomes 7A and 5B exhibited significant dominance of individual parents: 48.03% of 7A originated from Zhengmai 9405, while 4B269 and Zhoumai 16 contributed 23.62% and 28.35% of 7A, respectively. In 5B, Zhengmai 9405 and Zhoumai 16 each contributed 46.96% to Zhengmai 7698, whereas 4B269 contributed only 6.08%. Among the remaining chromosomes, only 1B, 2B, 2D, 3B, and 4A (Zhoumai 16) and 5A (Zhengmai 9405) had the genetic contribution of a parent more than 40% (Table S1).

The relative average genetic contributions (excluding 7A and 5B) of Zhengmai 9405, 4B269, and Zhoumai 16 were 32.3%, 30.6%, and 37.1%, respectively, and were therefore similar. Unlike previous reports^2–5^, this study did not find any single parent that contributed more than 60% of genetic material to any chromosome, which indirectly demonstrates that the frequency of recombination was higher in the multi-cross offspring than in the single-cross hybrids. Thus, the multi-cross offspring were more likely to experience pyramiding of superior alleles from its parents.

Zhengmai 7698 was selected using both conventional and molecular marker-assisted selection techniques. During the entire breeding process, agronomic traits were the first selection targets. The selected plants or lines with excellent agronomic traits were screened with markers, and those with a combination of desirable target genes were further selected for the next generation. Thus, selection efficiency was greatly improved because plants without target genes were excluded at early stages. In addition, new markers were added to the marker panel according to the requirements of breeding and cultivar approval authority. This study supports the extension of Zhengmai 7698 cultivation and can serve as a reference for the improvement of breeding methods for other wheat cultivars.

Due to the limitations of the wheat genome assembly and the gene annotation database, the 28,996 SNPs detected in this study and the 307 SNPs specific to Zhengmai 7698 could not be linked directly to agronomic traits. However, these SNPs could be used in other studies, such as trait linkage analyses, mapping of quantitative trait loci, and molecular marker-assisted breeding. Moreover, as Zhengmai 7698 is an important breeding parent with expanding cultivation in the southern Huanghuai wheat production area, the 28,996 high-quality SNPs identified here are more suitable for wheat genetic research of this region. Due to the commercialization of wheat breeding and the enhanced protection of breeder rights, the application of cultivar-specific markers to determine the authenticity and consistency of cultivars is becoming increasingly important, and the 307 SNP loci specific to Zhengmai 7698 identified here could be developed into specific markers for the accurate and rapid detection of Zhengmai 7698 cultivars.

## Conclusion

This study, using genome-wide high-density SNPs and gene-linked markers, revealed the genomic contribution of the three founder parents of the wheat cultivar Zhengmai 7698 and discovered 307 SNP loci specific to Zhengmai 7698. The characteristics of the genetic composition and structure of Zhengmai 7698 was also discussed. The results demonstrated that high-density SNP markers can be used to obtain a more precise evaluation of the genomic origin of cultivars.

## Materials and Methods

### Plant material

The cultivars used in this study were Zhengmai 7698, Zhengmai 9405, Zhoumai 16, and 4B269, which were provided by the Wheat Research Institute of the Henan Academy of Agricultural Sciences.

### Molecular markers

Molecular markers used in this study were mainly selected based on previous studies (Table 1). Traits included powdery mildew resistance genes, stripe rust resistance genes, pre-harvest sprouting resistance genes, and quality-specific genes (i.e., high molecular weight glutenin subunit, hardness, and yellow pigment content genes), comprising a total of 26 genes (Table 1).

### SNP marker development and whole genome scanning

SNPs implemented in this study were developed by Beijing Biomarker Technologies Co., Ltd. (Beijing, China) using specific-locus amplified fragment sequencing (SLAF-seq) as follows: (1) Following the prediction of restriction enzyme digestion reactions, suitable restriction endonucleases were selected to cleave genomic DNA from all qualified samples based on the optimal restriction digestion protocol. (2) The resulting fragments were subjected to 3ʹ-end adenylation and ligation with dual-index adapter sequences. Next, PCR amplification, purification, and mixing followed by gel extraction were performed to select the target fragments. After library quality was verified, an Illumina HiSeqTM2500 was used for paired-end sequencing at the average sequencing depth of 10X. (3) Raw sequence data were read for each sample using a dual-indexed library. After filtering out the adapter reads, the sequencing quality and amount of data were evaluated. (4) The efficiency of *Rsa*I digestion was evaluated using the control data to determine the accuracy and validity of the experiment. (5) The resulting genome-wide SNPs were identified using the genome sequence of the wheat cultivar Chinese Spring as the reference. SNPs of high-quality and high repeatability were selected for the further analysis.

To ensure the quality of the analysis, the quality score of the sequencing read used in this experiment was set to Q ≥ 30. A read length of 2 × 100 bp was used for subsequent data analyses and evaluation. The genomic sequence of the Chinese Spring cultivar was used as a reference for parametric analysis to obtain positional information and to annotate gene function of the sequenced fragments.

### SNP data analysis and genotype map construction

The resulting SNP genotypic data were used to analyse genomic background diversity in Zhengmai 7698 and its parents. For example, if the genotype of Zhengmai 7698 at a particular locus was the same as that of a specific parent but different from other parents, then Zhengmai 7698 was considered to have inherited that locus from that particular parent. Subsequently, the number of SNP loci contributed by each parent to the Zhengmai 7698 genome was calculated. The genetic transmission rate of a parent to Zhengmai 7698 was defined as the ratio of the number of SNP loci inherited from a particular parent to the total number of SNP loci. Based on this ratio, we obtained the genetic contribution of each of the three parental plants, Zhengmai 9405, 4B269, and Zhoumai 16, to Zhengmai 7698.

SNP loci on different chromosomes were sorted from the short arm to the long arm, according to the sequence alignment between the source sequences of the SNP loci and the wheat genomic sequence (Chinese Spring). An R-based programming script was used to construct the genotype map (provided by Beijing Biomarker Technologies Co., Ltd., China). Parental chromosomes were labelled with different colours based on the polymorphisms of their genotypes. Zhengmai 7698 was also labelled with different colours according to similarities and differences with its parents, which showed the genomic origin of this cultivar. Following a BLAST search of the SNP source sequences (E-value, 1e-5), the gene ontology (GO) database (www.geneontology.org) was used to perform a GO analysis and obtain gene annotations at the SNP loci.

### DNA extraction, PCR amplification, and detection of amplified products

Five seeds were selected from each cultivar, and leaves from each of these five plants were selected and pooled after sprouting. Genomic DNA was extracted using the CTAB (cetyltrimethylammonium Ammonium Bromide) method^9^, and the integrity and concentration of DNA extracts were tested using agarose gel electrophoresis (1%). The DNA was diluted to 100 ng/µL for marker detection. The PCR reaction volume of 12 μL included 2 µL DNA template, 1.2 µL 10 × buffer (Mg^2+^), 0.2 µL dNTPs (10 pmol/µL), 1.0 µL of the upstream (1 pmol/µL) and 0.38 µL of the downstream (10 pmol/µL) primer, 0.28 µL M13 primer (10 pmol/µL), 0.06 U Taq DNA polymerase (5 U/µL), and 6.88 µL ultrapure water. PCR amplification was carried out in a Mastercycler Pro S (Eppendorf AG, Hamburg, Germany). The PCR protocol consisted of an initial denaturation step at 95 °C for 5 min, followed by 35 cycles of denaturation at 94 °C for 30 s, annealing at 50 °C, 55 °C, or 60 °C (depending on the primers) for 30 s, extension at 72 °C for 45–60 s, and a final step at 72 °C for 10 min; the PCR products were then stored at 10 °C. Amplified products marked with sequence tagged site (STS) were analysed by conventional agarose gel electrophoresis (1–2%). SSR markers were detected by ABI 3730 quantitative fluorescence PCR, and KASP-SNP markers were detected by ABI7500 quantitative fluorescence PCR (Applied Biosystems, Foster City, CA, USA). Table 1 shows the details of the molecular markers used for PCR amplification.

## Electronic supplementary material

Supplementary information
