# Author Correction: Molecular marker assisted breeding and genome composition analysis of Zhengmai 7698, an elite winter wheat cultivar

**DOI:** 10.1038/s41598-019-51826-1

**Published:** 2019-10-17

**Authors:** Chun-xin Li, Wei-gang Xu, Rui Guo, Jian-zhou Zhang, Xue-li Qi, Lin Hu, Ming-zhong Zhao

**Affiliations:** Molecular Breeding Laboratory, Wheat Research Institute, Henan Academy of Agricultural Sciences, Zhengzhou, 450002 Henan China

Correction to: *Scientific Reports* 10.1038/s41598-017-18726-8, published online 10 January 2018

This Article contains errors in Table 1. In the HTML and PDF versions of this Article, the denotation of the genes used in F_3_-F_6_ generations, and F_7_ and subsequent stable generations is incorrect.

**Table 1:** Names and their primer sequences of markers used in the marker assisted selection of Zhengmai 7698. *: genes used in F_3_-F_6_ generations; **: genes used in F_7_ and subsequent stable generations; √: the existence of that gene. The primers of markers Pm2, Pm4b and Pm8 were presented, respectively, in Mohler et al. Theor Appl Genet 93: 1078–1082 (1996), Ma et al. Theor Appl Genet 109: 140–145 (2004) and Wang et al. Acta Genetica Sinica 28(7), 640–646 (2001).

Traits		Gene	Zhengmai 7698	Parents			Markers and their primers			Reference
Superior traits	High quality gene			4B269	Zhengmai 9405	Zhoumai 16	Marker	Forward primers (5′ - 3′)	Reverse primers (5′ - 3′)	
High molecular weight glutenin subunits		Ax1**	√	√			UMN19	CGAGACAATATGAGCAGCAAG	CTGCCATGGAGAAGTTGGA	10
		Ax-null**			√	√	UMN19	CGAGACAATATGAGCAGCAAG	CTGCCATGGAGAAGTTGGA	10
	Bx7*	Bx7*	√	√	√	√	Bx7	CACTGAGATGGCTAAGCGCC	GCCTTGGACGGCACCACAGG	11
	By8**	By8**		√	√		ZSBy8	TTAGCGCTAAGTGCCGTCT	TTGTCCTATTTGCTGCCCTT	12
		By9**	√			√	ZSBy9a	TTCTCTGCATCAGTCAGGA	AGAGAAGCTGTGTAATGCC	12
		Dx2**				√	UMN25	GGGACAATACGAGCAGCAAA	CTTGTTCCGGTTGTTGCCA	10
		Dx5*	√	√	√		Dx5	CGTCCCTATAAAAGCCTAGC	AGTATGAAACCTGCTGCGGAC	13
		Dy10**	√	√	√		UMN26	CGCAAGACAATATGAGCAAACT	TTGCCTTTGTCCTGTGTGC	10
		Dy12**				√	UMN26	CGCAAGACAATATGAGCAAACT	TTGCCTTTGTCCTGTGTGC	10
Grain hardness		Pinb-D1a*	√	√	√		Pinb-D1a	ATGAAGACCTTATTCCTCCTA	CTCATGCTCACAGCCGCC	14
		Pinb-D1b*				√	Pinb-D1b	ATGAAGACCTTATTCCTCCTA	CTCATGCTCACAGCCGCT	14
Lipoxygenase		*TaLox-B1a*	√		√		LOX16	CCATGACCTGATCCTTCCCTT	GCGCGGATAGGGGTGGT	15
		*TaLox-B1b*		√		√	LOX18	ACGATGTGAGTTGTGACTTGTGA	GCGCGGATAGGGGTGC	15
Yellow pigment content		Psy-A1a**				√	YP7A	GGACCTTGCTGATGACCGAG	TGACGGTCTGAAGTGAGAATGA	16
	Psy-A1b**	Psy-A1b**	√	√	√		YP7A	GGACCTTGCTGATGACCGAG	TGACGGTCTGAAGTGAGAATGA	16
		Psy-B1a**	√		√	√	YP7B-1	GCCACAACTTGAATGTGAAAC	ACTTCTTCCATTTGAACCCC	17
	Psy-B1b**	Psy-B1b**		√			YP7B-1	GCCACAACTTGAATGTGAAAC	ACTTCTTCCATTTGAACCCC	17
		*Psy1-D1g*		√	√		YP7D-1	TCCGACACCATCACCAAGTTCC	CGTTGTAGGTTTGTGGGAGT	18
Polyphenol oxidase activity		PPO-A1a**		√		√	PPO18	AACTGCTGGCTCTTCTTCCCA	AAGAAGTTGCCCATGTCCGC	19
	PPO-A1b**	PPO-A1b**	√		√		PPO18	AACTGCTGGCTCTTCTTCCCA	AAGAAGTTGCCCATGTCCGC	19
	PPO-D1a**	PPO-D1a**	√			√	PPO16	TGCTGACCGACCTTGACTCC	CTCGTCACCGTCACCCGTAT	20
		PPO-D1b**		√	√		PPO29	TGAAGCTGCCGGTCATCTAC	AAGTTGCCCATGTCCTCGCC	20
Powdery mildew resistant		Pm2*	√	√	√	√	Pm2	AGCTGTTTGGGTACAAGGTG	GCCATCGTTTTCTACTAG	21
		Pm4b*	√		√	√	Pm4b	GTGGTGTATCAAATGTCATCAGTACTAC	TCCAGTGACCCCATCTGCTCATAC	21
		Pm8*	√	√	√	√	Pm8	GGAGACATCATGAAACATTTG	CTGTTGTTGGGCAGAAAG	21
Yellow rust resistant		Yr9**	√	√		√	Xgwm582	AAGCACTACGAAAATATGAC	TCTTAAGGGGTGTTATCATA	22
		YrZH84**	√		√		Xcfa2040	TCAAATGATTTCAGGTAACCACT	TTCCTGATCCCACCAAACAT	23
Pre-harvest sprouting resistant		PHS1**	√	√		√	PHS1	GGTGGAACAGATGCAACTAAAGG/ GGTGGAACAGATGCAACTAAAGA	GTGAGTGTTATATGAAACTAATGATCCATT	24
		PHS-4AL**	√	√			PHS-4AL	TGGAGTCTGAAAGCATTCGA/ TGGAGTCTGAAAGCATTCGG	TCCATGCATCATAGGAAAACA	25

Additionally, the legend of Table 1 is incorrect:

“**Table 1**. Names and their primer sequences of markers used in the marker assisted selection of Zhengmai 7698 (the label √ means the existence of that gene).”

should read:

“**Table 1**. Names and their primer sequences of markers used in the marker assisted selection of Zhengmai 7698. *: genes used in F_3_-F_6_ generations; **: genes used in F_7_ and subsequent stable generations; √: the existence of that gene. The primers of markers Pm2, Pm4b and Pm8 were presented, respectively, in Mohler et al. Theor Appl Genet 93: 1078–1082 (1996), Ma et al. Theor Appl Genet 109: 140–145 (2004) and Wang et al. Acta Genetica Sinica 28(7), 640–646 (2001).”

The correct Table 1 and its accompanying legend appears below.

This Article contains errors in the Results section under subheading ‘Molecular markers for Zhengmei 7698’.

“In the F_3_–F_6_ generations of the selection processes, seven superior genetic markers in the parents were used (genes underlined by straight line in Table 1) to monitor the progress of gene pyramiding in hybrid offspring (e.g., single plant and line), and in the F7 and subsequent stable generations, and 19 newly developed genetic markers were progressively added (genes underlined by wavy line in Table 1).”

should read:

“In the F_3_–F_6_ generations of the selection processes, seven superior genetic markers in the parents were used (genes denoted with an * in Table 1) to monitor the progress of gene pyramiding in hybrid offspring (e.g., single plant and line), and in the F_7_ and subsequent stable generations, and 19 newly developed genetic markers were progressively added (genes denoted with an ** in Table 1).”

Furthermore, this Article contains errors in the Reference list. References 15, 18, 22 and 23 are incorrectly given as follows:

‘Geng, H. W., He, Z. H., Zhang, L. P., Qu, Y. Y. & Xia, X. C. Development of functional markers for a lipoxygenase gene on chromosome 4BS in common wheat. *Crop* Sci. **52**, 568–576 (2012).’

‘Wang, L. H. *et al*. Characterization of low-molecular-weight glutenin subunit *Glu-B3* genes and development of STS markers in common wheat (*Triticum aestivum* L.). *Theor. Appl. Genet*. **118**, 525–539 (2009).’

‘Weng, D. X. *et al*. Microsatellite marker linked with stripe rust resistant gene *Yr9* in wheat. *Acta Genetica Sinica* **32**, 937–941 (2009).’

‘Li, Z. F. *et al*. Molecular tagging of stripe rust resistance gene. *YrZH8425B. Theor. Appl. Genet*. **112**, 1098–1103 (2006).’

The correct references 15, 18, 22 and 23 appear below as references 1–4.
