# Supplementary material for: Molecular marker assisted breeding and genome composition analysis of Zhengmai 7698, an elite winter wheat cultivar
Source: Sci Rep. 2018 Jan 10;8:322. doi: 10.1038/s41598-017-18726-8 (PMC5762757; doi:10.1038/s41598-017-18726-8)
Supplement: Supplementary file 1 — Supplementary information [file 41598_2017_18726_MOESM1_ESM.pdf]

SUPPLEMENTARY INFORMATION

Manuscript title

Molecular marker assisted breeding and genome composition analysis of Zhengmai 7698, an elite winter wheat cultivar

Author list:

Chun-xin LI[1], Wei-gang XU*[1], Rui GUO1, Jian-zhou ZHANG[1], Xue-li QI[1], Lin HU[1] and Ming-zhong ZHAO[1]

[1]Molecular Breeding Laboratory, Wheat Research Institute, Henan Academy of Agricultural Sciences, Zhengzhou 450002, Henan, China

*Corresponding author: Weigang Xu (email: xuwg1958@163.com).

Content

Supplementary Figure S1. 1

Supplementary Figure S2. 2

Supplementary Figure S3...3

Supplementary Table S1 4

Supplementary Table S2.. 5

Supplementary Table S3.. 6

Supplementary Figure S1 Category analysis of the 146 genes identified by the SNP loci

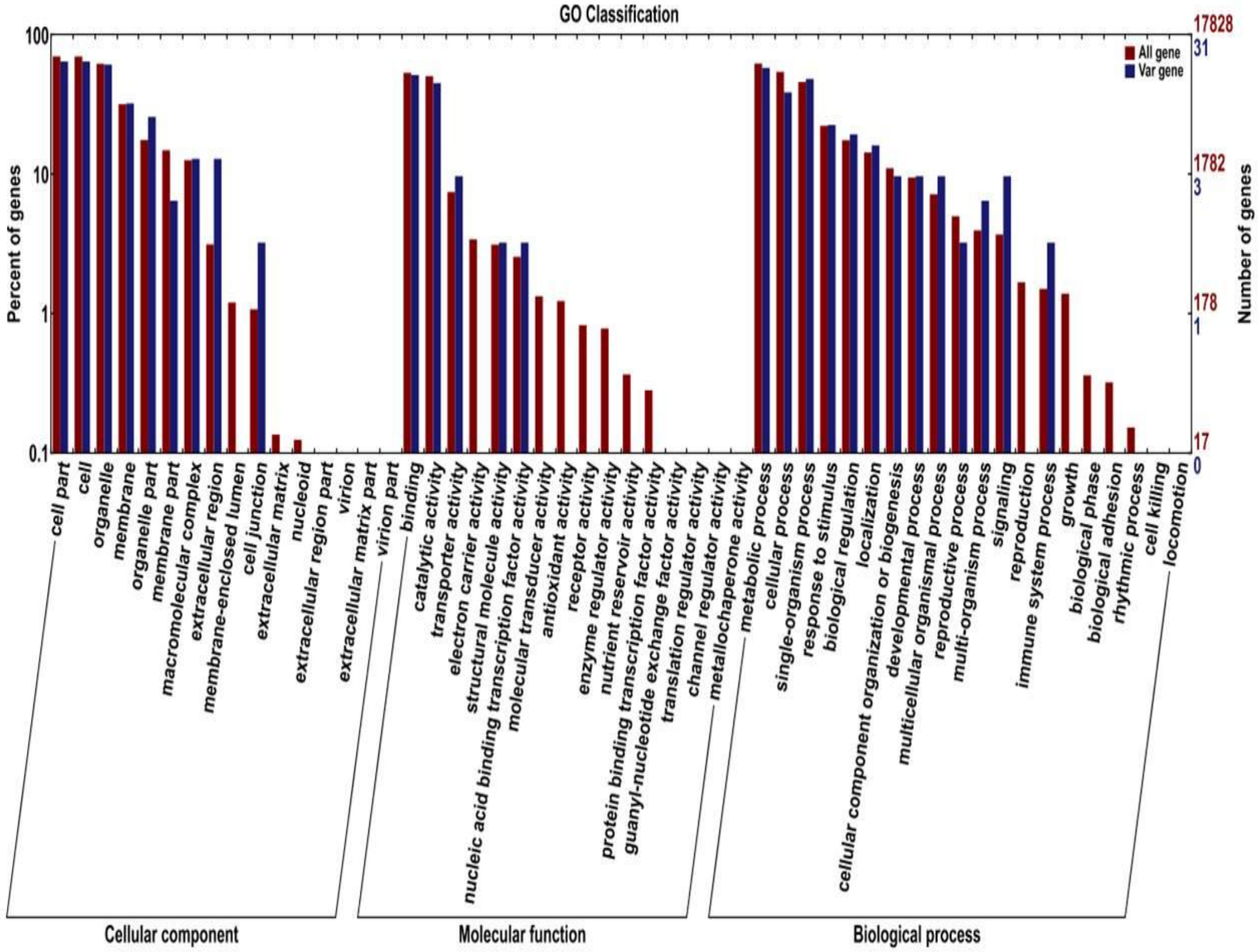

Supplementary Figure S2 Enrichment analysis in KEGG of the 146 genes identified by the SNP loci

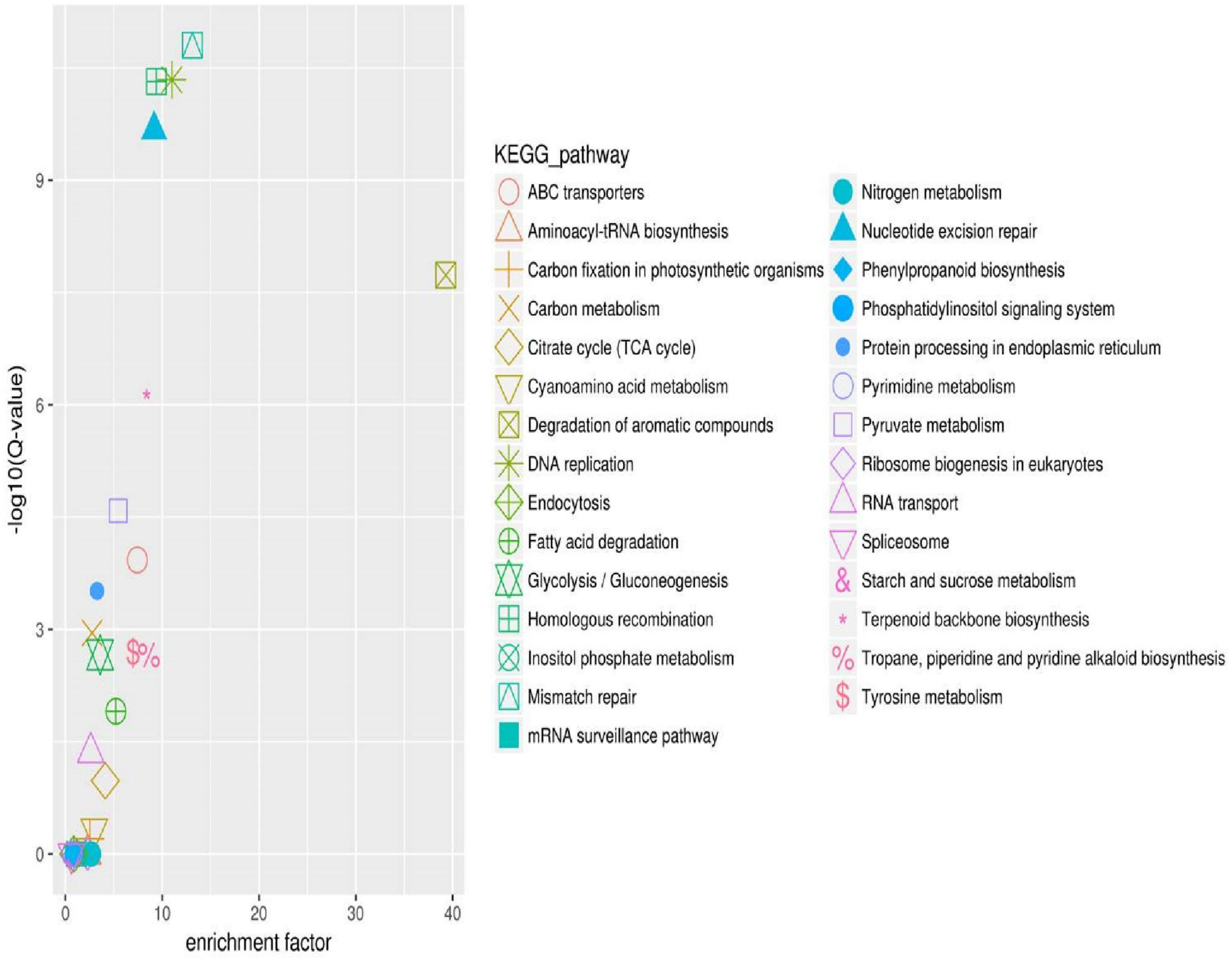

Supplementary Figure S3 Enrichment analysis in KEGG of the Zhengmai 7698's specific SNP loci

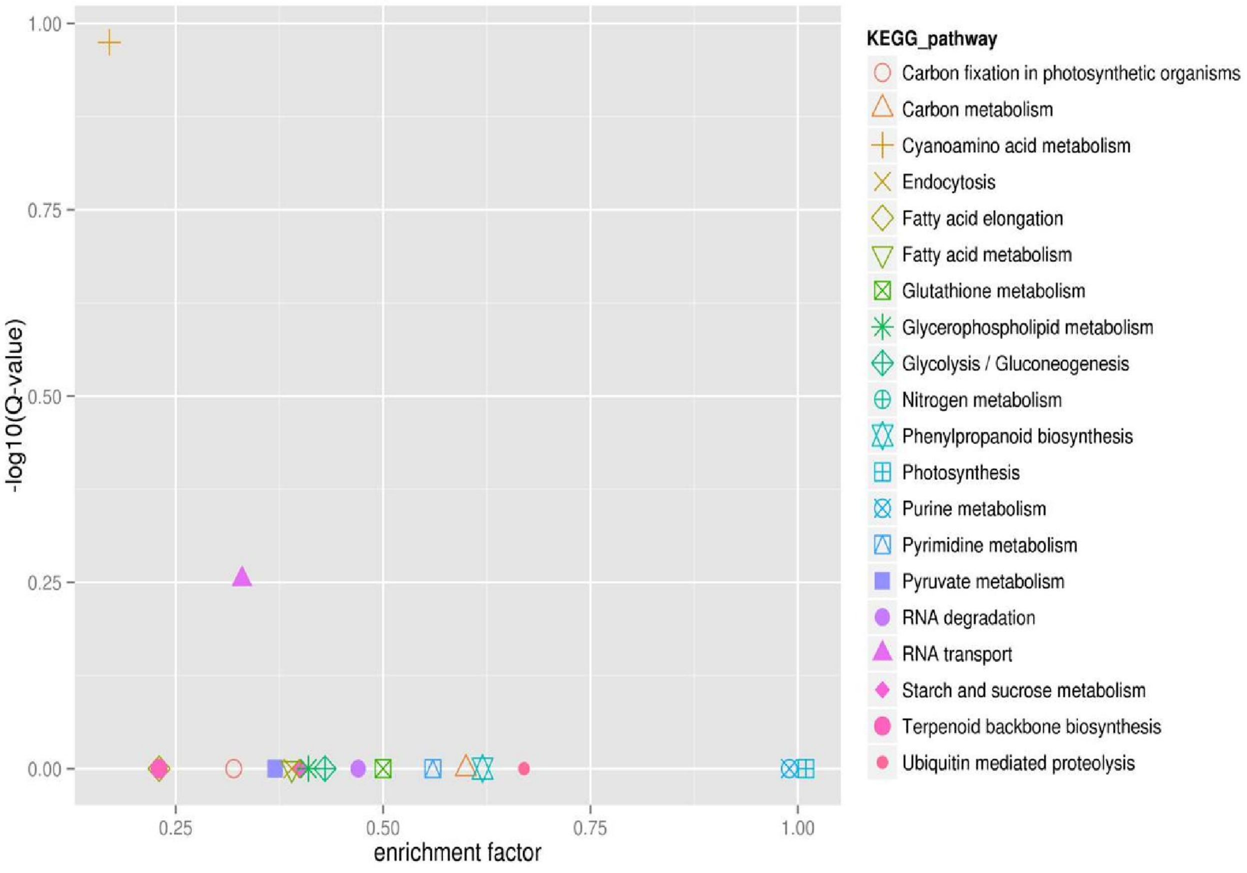

Supplementary Table S1 Relative genetic contribution ratio of three parents to Zhengmai7698

Chromosome	Zhengmai9405	4B269	Zhoumai16
1A	31.54%	34.62%	33.85%
2A	32.79%	31.15%	36.07%
3A	32.33%	29.32%	38.35%
4A	27.66%	31.21%	41.13%
5A	40.88%	24.53%	34.59%
6A	36.69%	28.78%	34.53%
7A	48.03%	23.62%	28.35%
1B	31.88%	22.50%	45.63%
2B	28.95%	28.29%	42.76%
3B	34.21%	25.00%	40.79%
4B	26.95%	34.75%	38.30%
5B	46.96%	6.08%	46.96%
6B	27.63%	34.21%	38.16%
7B	30.66%	37.23%	32.12%
1D	35.86%	33.79%	30.34%
2D	29.08%	28.37%	42.55%
3D	31.34%	32.84%	35.82%
4D	33.57%	29.37%	37.06%
5D	29.85%	32.84%	37.31%
6D	35.97%	30.94%	33.09%
7D	35.51%	31.88%	32.61%

Supplementary Table S2 Eight significant pathway of KEGG result of 28,996 SNPs loci

Pathway	KO	Enrichment_Factor	Q_value
Mismatch repair	ko03430	13.12	1.58E-11
DNA replication	ko03030	10.99	4.56E-11
Homologous recombination	ko03440	9.4	4.80E-11
Nucleotide excision repair	ko03420	9.16	2.11E-10
Degradation of aromatic compounds	ko01220	39.3	1.86E-08
Terpenoid backbone biosynthesis	ko00900	8.4	7.25E-07
Pyruvate metabolism	ko00620	5.45	2.60E-05
ABC transporters	ko02010	7.42	0.000118338

Supplementary Table S3 Gene Ontology result of specific SNP loci of Zhengmai7698

SNP Name	Position	Gene
1AL_3876943	8,018	Traes_1AL_206E50150.1
2BS_5242271	7,304	Traes_2BS_FB140D3A3.1
2BS_5242271	7,304	Traes_2BS_9957A14D4.1
2DS_5366734	3,693	Traes_2DS_A3D068B05.1
2DS_5366734	3,739	Traes_2DS_A3D068B05.1
3B_10480957	258	Traes_3B_A934CAE47.1
5BS_2239251	18,024	Traes_5BS_2FEE2EAFF.1
5BS_2239251	18,024	Traes_5BS_805D85BDE.1
5BS_2239251	18,024	Traes_5BS_CC67ACF49.1
5BS_2239251	18,024	Traes_5BS_03BBAB2C2.1
5DL_4524326	5,209	Traes_5DL_FEC3DC25B.1
7AL_4449824	999	Traes_7AL_8A07184BC.1
7AL_4556119	932	Traes_7AL_91A8A5DC5.1
